# Supplementary material for: Polymorphism rs259983 of the Zinc Finger Protein 831 Gene Increases Risk of Superimposed Preeclampsia in Women with Gestational Diabetes Mellitus
Source: Int J Mol Sci. 2024 Oct 16;25(20):11108. doi: 10.3390/ijms252011108 (PMC11508172; doi:10.3390/ijms252011108)
Supplement: Supplementary file 1 [file ijms-25-11108-s001.zip › ijms-3213813-supplementary.pdf]

Supplementary Table S1. Genotype distribution, allele frequency, and p-value of Hardy-Weinberg equilibrium for PE+ and Control groups in pregnant women without CHTN

Sample type	Genotype distribution, n (%)			Allele frequency		p-value
	AA	AC	CC	A	C	
**PE+, n=147**	105 (71.43)	39 (26.53)	3b (2.04)	0.85	0.15	1
**Control, n=198**	132 (66.67)	60 (30.3)	6 (3.03)	0.82	0.12	1

Supplementary Table S2. *Association between rs259983 of the ZNF831 gene and preeclampsia in pregnant women without CHTN*

Gene/SNP	Model of inheritance	Genotypes	PE+, n=147	Control, n=198	OR (95% of CI)	p-value[1]	AIC
ZNF831 rs259983	Codominant	AA	105 (71.4)	132 (66.7)	1.00	0.6	475.7
		AC	39 (26.5)	60 (30.3)	0.82 (0.51-1.32)		
		CC	3 (2.0)	6 (3.0)	0.63 (0.15-2.57)		
	Dominant	AA<br>AC + CC	105 (71.4)<br>42 (28.6)	132 (66.7)<br>66 (33.3)	1.00<br>0.8 (0.5-1.27)	0.34	473.8
	Recessive	AA + AC<br>CC	144 (98.0)<br>3 (2.0)	192 (97.0)<br>6 (3.0)	1.00<br>0.67 (0.16-2.71)	0.56	474.4
	Overdominant	AA + CC<br>AC	108 (73.5)<br>39 (26.5)	138 (69.7)<br>60 (30.3)	1.00<br>0.83 (0.52-1.34)	0.44	474.1
	log-Additive	0, 1, 2	147 (42.6)	198 (57.4)	0.81 (0.54-1.22)	0.31	473.7

[1]Statistically significant result with p-value < 0.05

Supplementary Table S3. Genotype distribution, allele frequency, and p-value of Hardy-Weinberg equilibrium for PE+ and Control groups in pregnant women without CHTN or obesity

Sample type	Genotype distribution, n (%)			Allele frequency		p-value
	AA	AC	CC	A	C	
**PE+, n=81**	51 (62.96)	27 (33.33)	2 (2.7)	0.80	0.20	1
**Control, n=147**	99 (67.35)	42 (28.57)	6 (4.08)	0.82	0.18	0.58

Supplementary Table S4. Association between rs259983 of the ZNF831 gene and preeclampsia in pregnant women without CHTN or obesity

Gene/SNP	Model of inheritance	Genotypes	PE+, n=81[1]	Control, n=147	OR (95% of CI)	p-value[2]	AIC
ZNF831 rs259983	Codominant	AA	51 (63.0)	99 (67.3)	1.00 (References)	0.76	302.1
		AC	27 (33.3)	42 (28.6)	1.25 (0.69-2.25)		
		CC	3 (3.7)	6 (4.1)	0.97 (0.23-4.04)		
	Dominant	AA<br>AC + CC	51 (63.0)<br>30 (37.0)	99 (67.3)<br>48 (32.7)	1.00<br>1.21(0.69-2.14)	0.51	300.3
	Recessive	AA + AC<br>CC	78 (96.3)<br>3 (3.7)	141 (95.9)<br>6 (4.1)	1.00<br>0.9 (0.22-3.71)	0.89	300.7
	Overdominant	AA + CC<br>AC	54 (66.7)<br>27 (33.3)	105 (71.4)<br>42 (28.6)	1.00<br>1.25 (0.7-2.24)	0.46	300.1
	log-Additive	0, 1, 2	81 (35.5)	147 (64.5)	1.13 (0.7-1.83)	0.61	300.4

[1]PE+ groupe with excluded women with CHTN or obesity

[2]Statistically significant result with p-value < 0.05

Supplementary Table S5. Genotype distribution, allele frequency, and p-value of Hardy-Weinberg equilibrium for PE+ and SIPE groups in pregnant women

Sample type	Genotype distribution, n (%)			Allele frequency		p-value[1]
	AA	AC	CC	A	C	
**PE+, n=147**	42 (61)	18 (26)	9 (13)	0.74	0.26	0.01
**SIPE, n=69**	105 (71)	39 (27)	3 (2)	0.85	0.15	1

[1]Statistically significant result with p-value < 0.05

Supplementary Table S6. Association of the rs259983 of the ZNF831 gene between PE+ and SIPE in pregnant women

Gene/SNP	Model of inheritance	Genotypes	SIPE, n=69	PE+, n=147	OR (95% of CI)	p-value[1]	AIC
ZNF831 rs259983	Codominant	AA	42 (60.9)	105 (71.4)	1.00 (References)	0.006	266.5
		AC	18 (26.1)	39 (26.5)	1.15 (0.59-2.24)		
		CC	9 (13.0)	3 (2.0)	7.5 (1.94-29.07)		
	Dominant	AA<br>AC + CC	42 (60.9)	105 (71.4)	1.00<br>1.61 (0.88-2.93)	0.12	272.3
	Recessive	AA + AC<br>CC	27 (39.1)	42 (28.6)	1.00<br>7.2 (1.88-27.52)	0.002	264.7
	Overdominant	AA + CC<br>AC	60 (87.0)	144 (98.0)	1.00<br>0.98 (0.51-1.87)	0.94	274.6
	log-Additive	0, 1, 2	9 (13.0)	3 (2.0)	1.81 (1.13-2.91)	0.01	268.5

[1]Statistically significant result with p-value < 0.05

Supplementary Table S7. Flanking sequences for rs259983 of the ZNF831 gene (± 400 base pairs up- and down-stream from polymorphism)

>20 dna:chromosome chromosome:GRCh38:20:59160002:59160802:1

GCTGGATGCTGGGTTCCATTGTGAGGCCAGGCACAGCCAAGGCAGAGACGAACATTCCTACA
GTCGCTCACGACACCAAGCTCCACAGACCAGAGAATGGACGGATCCACAGGAACGAAAGAC
AGGCTGTCAGGTGCCCAGCCCTGGGGAGACTCCAGAAGAGGAGAGAGGACCACCCATCATG
GCTGACCTTGGGAGGACTTGGGTCTTCACAAATTGTGTTCCTTGGTATATATAAGAGGGTTAA
AATAGACACAAGAAATATCCATCTATTCCCATTCATTTATTTGACACATACTCTTGGGGCCAT
GAGTTCAATTCCCAGAAGAGGATCATGATGGTGGGGGGAGGAAGGATGTGGCGAGGGGCCG
TGCAGGAGCCCAGGGGGCTTGTCTCATGGACGCTCTTGATCGAATTCCTCTGAGGCCCCAGA
GGTGCTCCTTCATCCCCAGCTTGCTTGTGAGGCTGCGTTGGCTCCCCAAGGCCGAGTTTCCTGC
TGAGACCCAGCCACACACTTAGCCCTGAGTGTACCAGGTGGCTCCACTCAGAGCGCTGCTCC
AGCCCCTCTCCCTGGGGCTTCCTGCCACGTGACTGCACATTTGGGGTCTGGAGAAGGGCCCAG
GGACTGAACTGCAAAGGTGAAGGGTGTCCCTGCTCCTCCTGACCACAGCTTCTGGACTGGCA
TGTGTGTCTGATGGTGCAAGACCAGAGGGGCATGGCCAATGAGAGAGTTATTCTTTCTACCCG
TGAGTCTCCCTCGGACACACAGGATTTCTCATGTCTCCTCCTGGTTTTGCTGCT

Supplementary Method S1. *Blood samples collection, transportation and storage*

Biological blood samples were obtained by drawing blood from the cubital vein, immediately before birth. Blood in a volume of 5 ml was collected into sterile tubes with 50 µl of 0.5 M EDTA, pH=8.0, which was used as an anticoagulant. After collection, samples were stored at +4°C and transported to the laboratory at the same temperature. After entering the laboratory for the regulation of reparative processes, genomic DNA (gDNA) from venous blood samples was isolated using phenol-chloroform extraction according to Maniatis, followed by precipitation of gDNA with ethanol and elution with TE buffer (Evrogen). Qualitative and quantitative assessment of isolated DNA was carried out on a NanoDrop 1000 spectrophotometer in dsDNA-50 (double-stranded DNA) analysis mode. The analysis was carried out in NanoDrop 1000 Operating Software version 3.8.1. gDNA was stored at -20°C until genotyping.
